# *In vivo* oxidative stress associated with growth-related myopathies in chicken and potential health impact: an opinion paper

**DOI:** 10.3389/fphys.2023.1291323

**Published:** 2023-11-02

**Authors:** Yuwares Malila

**Affiliations:** Food Biotechnology Research Team, National Center for Genetic Engineering and Biotechnology, Pathum Thani, Thailand

**Keywords:** broilers, chicken meat, myopathy, protein oxidation, oxidative stress

## 1 Introduction

Chicken meat, particularly the breast portion, offers high-quality protein, providing adequate amounts of all of the essential amino acids with a Protein Digestibility Corrected Amino Acid Score value ranging between 0.91 and 0.95 (Boye et al., 2012). Among land animal meats, chicken breast is fairly low in lipid and collagen which is more favorable for protein digestibility (Barrón-Hoyos et al., 2013; Marangoni et al., 2015). Global demand for poultry meat has been steadily rising and it has been projected at 145 tons by 2029, with chicken expected to account for 50% of the meat consumed (USDA, 2021). A steady increase in chicken breast meat consumption within the next decade has been predicted although alternative proteins, e.g., insect and plant-based proteins, has gained increasing attention. The main reason behind its high demand is its affordable price for all classes of consumers. In addition, for the past decades, meat consumption has shifted from predominantly red meat to white meat as a healthier choice.

For the past decades, commercial broilers have been intensively selected through a breeding selection for production efficiency to meet high consumer demand (Barbut and Leishman, 2022). The success in breeding selection, however, has coincided with increased abnormalities among broilers, including growth-related myopathies, namely, White striping (WS), Wooden breast (WB) and Spaghetti meat (Barbut, 2019; Petracci et al., 2019; Soglia et al., 2019; Barbut and Leishman, 2022). These myopathies can be found together or individually in all broiler chicken breeds with a large variation in occurrence and severity across global regions (Lorenzi et al., 2014; Malila et al., 2018; Barbut, 2019; Soglia et al., 2019; Che et al., 2022).

The issue of growth-related myopathies in broilers are globally recognized among poultry community. The industry has found an increasing prevalence of those abnormalities in the past decade with mild WS as the “new norm” of chicken breast meat. Originally found only in the breast (*Pectoralis major*), occurrence of WS and WB has now been observed in other cuts, including chicken filet (*Pectoralis minor*) and thigh (Petracci et al., 2019). A number of studies previous investigated the approaches, including selecting the hybrids with slower growth rate (Gratta et al., 2019), slowing the growth with manipulating the amino acids in the feed (Meloche et al., 2018; Zampiga et al., 2019; Lackner et al., 2022), and terminating the birds at the younger ages (Abreu et al., 2022) with an attempt to reduce the prevalence of the myopathies. Although the previous studies addressed the experimental reduction of the myopathies, the issue at the industrial scale does still exist.

## 2 An association between growth-related myopathies in broilers and occurrence of *in vivo* oxidative stress

The actual etiology of the myopathy is still under investigation. Yet, previous histological studies consistently show chronic muscle fiber damage as shown by accumulated macrophages, large-rimmed vacuoles, nuclei internalization, deposition of adipocytes, thickened endomysium and perimysium, inconsistent size of rounded myofibers, infiltration of lymphocytes and macrophages, and necrosis in the affected muscles (Kuttappan et al., 2012; Sihvo et al., 2014; Papah et al., 2017; Malila et al., 2018; Salles et al., 2019; Hosotani et al., 2020; Praud et al., 2020). The occurrence of fiber necrosis, fibrosis and adipose tissue filtration increased as the severity degree of the myopathies increased (Praud et al., 2020). The muscle fiber damage has been hypothesized as an adverse consequence when the muscle fibers were outgrown their supportive systems, particularly vascularization (Mutryn et al., 2015; Alnahhas et al., 2016; Kindlein et al., 2017; Papah et al., 2017; Sihvo et al., 2018; Lake and Abasht, 2020). In male Cobb 500 broilers, an increased intercapillary distances together with reduced ratio of capillary to muscle fibers were correlated with WB severity level (Kindlein et al., 2017). The early pathogenesis of WB was likely associated with endothelial cell dysfunction, particularly in the capillaries and venous ends of the vasculature (Abasht et al., 2021). In the breast muscle of commercial broilers, multifocal perivascular and perivenous aggregates of lipid-laden macrophages were observed at 1 week of age prior to the development of myopathic lesions at 2 weeks of age (Papah et al., 2017). Limited oxygenation (Kindlein et al., 2017) in combination with the sequellae of phlebitis and impaired venous drainage (Papah et al., 2017; Abasht et al., 2021) may lead to local accumulation of metabolic waste and reactive oxygen species (ROS), triggering muscle fiber degeneration. In addition, lipid accumulation in the affected *Pectoralis major*, resemblance to type 2 diabetes, may exert cellular stress to the cells and further suppress glycolysis and gluconeogenesis in the affected birds (Lake and Abasht, 2020). Accumulated lipids can enhance oxidative stress through the lipid peroxidation of fatty acids (Li et al., 2022). In addition, ROS can readily react with other biomolecules, particularly lipids, proteins and DNA (Min and Ahn, 2005). Malondialdehyde, a product of lipid oxidation, has been shown to be responsible for secondary carbonylation of myoglobin and myofibrillar proteins along with cross-linking of myofibrillar proteins from rabbit skeletal muscle (Wang et al., 2020; Yin et al., 2022).

Differential gene expression patterns associated with development of growth-related myopathies suggested alteration of several biological processes, including metabolisms of nutrients, programmed cell death, to muscle regeneration (Mutryn et al., 2015; Zambonelli et al., 2016; Malila et al., 2020). Among the key stress-related transcription factors, transcript abundance of hypoxia-inducible factor 1 (HIF-1), particularly alpha subunit (HIF1A), along with antioxidant enzymes, particularly superoxide dismutase isoform 2 and 3, were increased in the myopathic muscles (Malila et al., 2019; Marchesri et al., 2019; Malila et al., 2022). The findings implied molecular activities against cellular oxidative stress. However, chronic hypoxia within the affected breast muscle appeared to weaken HIF signaling and disrupt the processes of autophagy and mitophagy (Hosotani et al., 2020). In turn, such pathological condition attenuated adaptability of the muscle to hypoxia. The stress environment might trigger aberrant activity of fibro-adipogenic progenitors, resulting in fibrosis (Malila et al., 2022). Metabolic intermediates, i.e., fumarate, and malate, from tricarboxylic acid cycle, were accumulated in the affected breast muscles, suggesting the back flux of oxaloacetate converted into malate and fumarate under limited oxygenation condition (Boerboom et al., 2018). An increased conversion of L-arginine into citrulline was observed in WS breast muscle, presumably to produce nitric oxide (Boerboom et al., 2018). When the level of nitric oxide was elevated, tyrosine residue on polypeptide chains could undergo oxidation, resulting in nitrotyrosine which was associated with inflammation diseases (Cai and Yan, 2013).

## 3 Potential effects of consuming oxidized lipids and proteins

Although whether consumption of oxidized lipids and proteins would cause any chronic diseases in human is still inconclusive, a growing evidence demonstrated that diets containing excessive oxidative products showed potential to disturb *in vivo* cell redox status (Estévez and Luna, 2017; Estévez and Xiong, 2019; Hu et al., 2021). The chronic oxidative stress is not only responsible for the virulence and severity of the disease but also the oxidative DNA damage of the epithelial cells (Hardbower et al., 2013). Damages of DNA may interrupt transcription leading to the aberrant cellular response to the oxidative stress or the obtain of erroneous protein structure and functions (Huang and Anh, 2019). Unless the damage to DNA is repaired, it can induce long-term physiological conditions, including inflammation, atherosclerosis, aging and cancer (Huang and Anh, 2019). The intake of high-fat diets and oxidized lipids has been known to be associated with pathological conditions (Estévez and Xiong, 2019). Previous studies in animal models suggested that consumption of diet containing oxidized oils elevated the risk of cellular oxidative stress (Dalle-Donne et al., 2006). An association between 4-hydroxyhexenal, a lipid peroxidation product, and the progression of Alzheimer’s disease was addressed (Bradley et al., 2012).

In contrast to oxidized lipids, the investigation regarding impacts of consuming dietary oxidized proteins have recently gained attention (Estévez and Xiong, 2019). As reviewed by Soladoye et al. (2015), Estévez and Xiong, (2019), and Domínguez et al. (2022), an accumulation of oxidized proteins and their products (e.g., heterocyclic aromatic amines or advanced-glycation end products) was linked with pathological conditions of certain diseases (e.g., Parkinson’s, Alzheimer’s, type II diabetes, and renal failure). Protein carbonylation, an irreversible modification associated with oxidative damage, has been widely used as a biomarker for protein oxidation (Cai and Yan, 2013). The modification occurs on multiple amino acid residues on selected protein targets, including arginine, histidine, lysine, proline, threonine and cysteine (Soladoye et al., 2015). Hence, quantity and quality of essential amino acids were reduced (Soladoye et al., 2015). Gut proteases may not recognize the target sites on the oxidized proteins, leading to reduced protein digestibility and bioavailability (Soladoye et al., 2015). Protein carbonyls may induce polymerization among the oxidized proteins or between their derivatives and other polypeptide chains. On the contrary, in severe condition, the carbonyls can also attack the peptide backbone, resulting in breakdown of the polypeptides into several carbonyl-containing peptides (Stadtman and Levine, 2003). In addition, lipid-derived protein carbonyls can promote a pro-oxidative environment in the muscle tissue. An example was the role of 4-hydroxynonenal in the formation of formation of protein adducts in heart, liver and skeletal muscle of rats (Keller et al., 2020).

## 4 Health consequences of consuming growth-related myopathies in chicken breast meat: should it be concerned?

**FIGURE 1:**
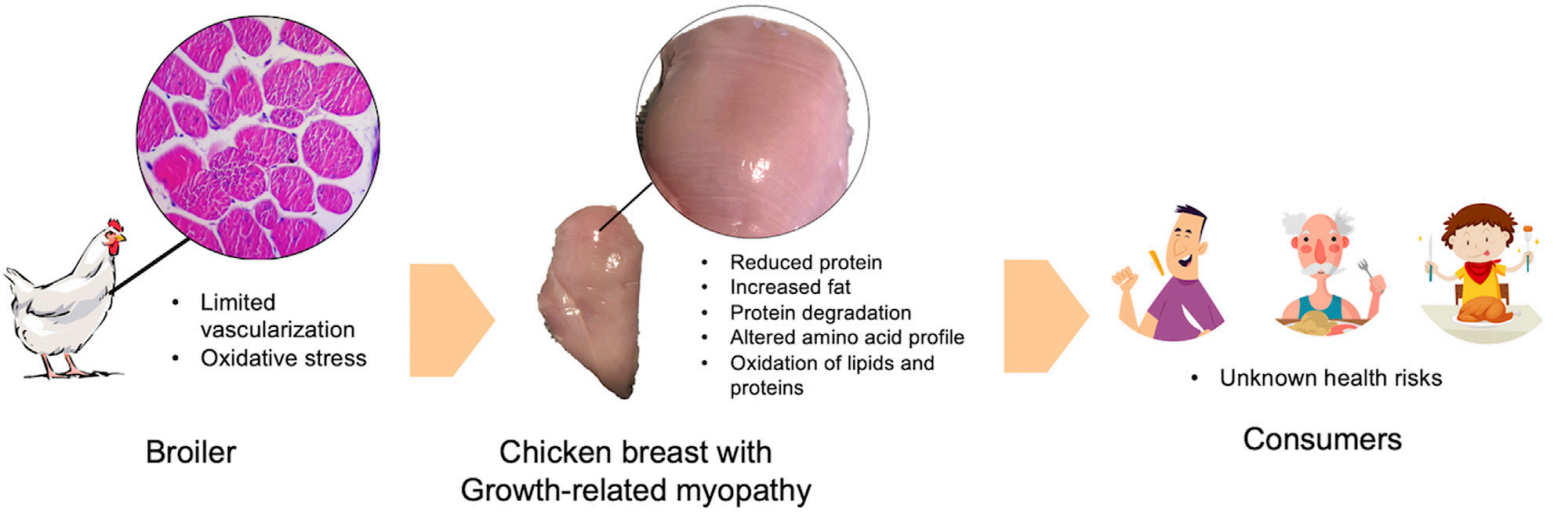
A schematic diagram depicts an association between growth-related myopathies and oxidative stress. Impacts of growth-related myopathies and the assumption of health consequence are included.

Previous studies consistently demonstrated a decreased proportion of protein and increased fat in raw chicken breast meat severely affected with WS and WB condition (Petracci et al., 2014; Soglia et al., 2016; Baldi et al., 2018; Malila et al., 2018; Soglia et al., 2018; Adabi and Suncu, 2019; Mudalal, 2019; Carvalho et al., 2021). The deviation of chemical composition can shift energy distribution of the affected chicken breast meat towards energy contribution from fat (Petracci et al., 2014). The change was more pronounced as the severity elevated. Profile of essential amino acids were also altered in the myopathic chicken meat (Adabi and Suncu, 2019; Soglia et al., 2019; Dalle Zotte et al., 2020; Thanatsang et al., 2020). Proteins might undergo degradation into free amino acids in the affected breast (Soglia et al., 2019) which might be potentially lost with dripping and purging fluids during storage and cooking, respectively. In addition, antemortem oxidative stress condition would result in increased ROS and free radicals promoting oxidation of lipids and proteins in food (Estévez and Xiong, 2019). Given that growth-related myopathies were associated with oxidative stress (Thanatsang et al., 2020; Li et al., 2022), one may assume that when chicken meat with high severity of WS and WB abnormalities was consumed, consumers might increase risk of an expose to oxidized lipids and proteins (Figure 1). However, no investigation has been conducted on such aspect.

So far, because the issue of growth-related myopathies has been emerged for about a decade, the published research has been emphasized on the impacts on technological properties and underlying their etiology with the best attempt to establish effective solutions. Apart from those aforementioned altered macronutrients, other health consequences due to consumption of those growth-related myopathies have not been investigated. Whether such altered macronutrients would significantly exert health impacts remains unclear. It is worth noting that in most countries, the severe WB are rejected and only focal mild cases are utilized as human food. Additionally, muscle damages were widely detected on the superficial part of the affected breast and the lesions were less pronounced at the deeper regions (Sihvo et al., 2014; Soglia et al., 2017; Baldi et al., 2018); hence, it is reasonable to hypothesize that consumption of the whole breast meat may less likely exert any adverse health consequences. However, media and internet began to criticize chicken breasts with growth-related myopathies. Such information may someday gain wide attention and eventually exert any negative perception and fear towards chicken meat and poultry industry. Therefore, it is essential that scientific community begins to gather the reliable scientific evidence regarding the influence of growth-related myopathies on protein quality, protein bioavailability and health impact particularly among susceptible consumers (e.g., elderlies and patients) at a long-term exposure.
